# Contamination by Potentially Toxic Elements (PTEs) in Agricultural Products Grown Around Sepetiba Bay, Rio de Janeiro State (SE Brazil)

**DOI:** 10.1007/s00244-025-01143-8

**Published:** 2025-08-06

**Authors:** Graziele Arantes Reis, Maria Virginia Alves Martins, Lisia Maria Gobbo Santos, Santos Alves Vicentine Neto, Fernando Barbosa Junior, Mauro Cesar Geraldes, Sérgio Bergamaschi, Rubens Cesar Lopes Figueira, Carla Patinha, Eduardo Ferreira da Silva, Fernando Rocha

**Affiliations:** 1 Universidade do Estado do Rio de Janeiro, UERJ, Av. São Francisco Xavier, 524, Maracanã, Rio de Janeiro, RJ Zip Code: 20550-013 Brazil; 2 Departamento de Geociências, Universidade de Aveiro, GeoBioTec, Campus de Santiago, 3810-193 Aveiro, Portugal; 3 Departamento de Química, Instituição Nacional de Controle em Qualidade em Saúde, Fundação Oswaldo Cruz, Av. Brasil, 4365 - Manguinhos, Rio de Janeiro, RJ Zip Code: 21.040-900 Brazil; 4 Faculdade de Ciências Farmacêuticas de Ribeirão Preto (FCFRP), Laboratório de Toxicologia Analítica e de Sistemas, Universidade de São Paulo, USP, Av. Professor Doutor Zeferino Vaz, s/n, Ribeirão Preto, São Paulo, SP Zip Code: 14040-903 Brazil; 5 Instituto Oceanográfico, Universidade de São Paulo (IOUSP), Pça. do Oceanográfico, 191, Butantã, São Paulo, Zip code: 05508-120 Brazil

## Abstract

The contamination of soil, water, and air by potentially toxic elements (PTEs) allows the transfer of metals to food. When introduced into food through soil, PTEs are absorbed by plant roots and transported to the bloodstream. The region surrounding Sepetiba Bay (SB), a densely populated area intensively exploited for tourist purposes, is one of the most contaminated by PTEs in the southeastern region of Brazil. Therefore, this study aims to evaluate (by ICP-MS) the concentrations of PTEs in agricultural products (roots, fruits, leaves, seeds, cheese, and eggs) cultivated and sold by rural producers in the region surrounding SB. The 26 food types selected for this study were produced in this region and purchased at local markets. The results for the analyzed foods revealed relatively high concentrations of Zn > Cu > Ni > Cr > As > Pb > Co > Hg > Cd, exceeding the limits established by ANVISA and international laws. Cluster analysis (CA) and principal component analysis (PCA) grouped the PTEs according to their possible sources: ore transport (Zn, Cu, Pb, and Cd), natural (Hg, Cr, and Ni), and fertilizers (Co and As). The CA allowed us to distinguish two main food groups in terms of the enrichment of PTEs. Group 1 contains leaves, animal products, and seeds with higher levels of Hg, Cr, Ni, Co, and As. Group 2 includes roots and fruits, which have higher concentrations of Zn, Cu, Pb, and Cd than Group 1. The Hazard Quotient (HQ) suggests that As and Cr pose a higher risk in foods of CA-Group 1 (leaves, animal products, and seeds). Hazard Index (HI) values > 1 indicate a risk of probable long-term carcinogenesis in adults. Thus, this study reveals that the continued consumption of food produced in the region surrounding SB could cause health problems for the local population.

**Supplementary Information:**

The online version contains supplementary material available at 10.1007/s00244-025-01143-8.

Potentially toxic chemical elements (PTEs) occur naturally, composing the mineral structure in rocks, soils and sediments and can be grouped into essential (e.g., Cu, Fe, Mn, Ni, Zn) when they constitute soil nutrients and play a role in the living beings and non-essential (e.g., As, Cd, Pb, Ni, Hg) when they present a risk to the organisms, even at low concentrations (Shaheen et al. 2016; Ghosh et al. 2020). PTEs are weather-resistant and are among the substances that may present the most severe risk due to their degree of toxicity (Rahman et al. 2018; Chakraborty et al. 2022).

Weathering and erosion generate deterioration of rocks and sediments, facilitating the dispersion of PTEs in the environment. Some anthropogenic activities accelerate the availability of PTEs in waters and soils (Gupta et al. 2019). Climatic factors and temperature variations influence the dispersion of PTEs in the soil (Pourghasemian et al. 2013; Cornu et al. 2016), as well as the pedogenesis and the degree of maturation of the products at the time of harvesting (Khan et al. 2008), as well as anthropogenic actions.

Intensive agricultural and industrial activities, as well as gas emissions and domestic effluents, are among the primary sources of soil contamination (Adimalla et al. 2019; Sun et al. 2019). These factors contribute to the spread of pollutants in the environment, and metal contamination can affect the health of living organisms.

Plant roots are the first tissues to absorb the PTEs available in the soil, which are later translocated to the stem, leaves, fruits, and seeds (Meng et al. 2014; Zhang et al. 2016; Gupta et al. 2019). The PTE concentrations in vegetables are, naturally, low. However, in areas where intense industrial, port, and mining activities occur, PTE enrichment can increase considerably (Khan et al. 2018).

In these areas, the accumulation of PTEs can modify the essential functions of plant maturation and survival (Agrawal et al. 2007; Singh et al. 2016). Heavy metals are concentrated in different proportions in vegetables and fruits (Ruzaidy and Amid 2020; Lemessa et al. 2022). Agricultural products play a crucial role in the human diet, both in raw and cooked forms (Jolly et al. 2013). Santhiravel et al. (2022) emphasized the role of plants in supporting the functioning of the human body, including their contribution to the production of acids during digestive processes. However, when they have high PTE contents, they can pose a risk to human health.

PTEs can bioaccumulate and biomagnify in living beings, including humans (Alexander 1999; Hare 2013; Zakir et al. 2020; Zhang et al. 2021; Saidon et al. 2024) through the food chain (Jolly et al. 2013). Ingestion of contaminated food is among the most common routes of contamination by PTEs (Vardhan et al. 2019). Biomagnification is understood as a process of metal transfer in the food web, considering trophic levels and consumer position (Gall et al. 2015). Thus, there is a gradual accumulation of PTEs along food chains and webs, primarily affecting organisms at higher trophic levels (Gall et al. 2015; Soliman et al. 2019).

The degree of toxicity of PTEs concerning human health depends on the quantity, duration of exposure, chemical and physical properties of the metals, and the route of contamination (Santos et al. 2017). PTEs have a high degree of toxicity to the human body (Kim et al. 2021). Among the adverse effects that PTEs can trigger in the human body are neurotoxicity, carcinogenesis, teratogenesis, and changes in cellular oxidation (El-Kady and Abdel-Wahhab 2018).

The ingestion of food contaminated by PTEs can generate human nervous system disorders, dementia, insomnia, and liver and eye dysfunctions (Emamverdian et al. 2015; Jan et al. 2011). In addition, on more severe occasions, when they attach to cells, they can cause carcinogenic risks (Smith et al. 2000).

Studies conducted in the coastal zone located in the southern part of the State of Rio de Janeiro reveal high levels of environmental pollution by potentially toxic elements (PTEs) in sediments, water, plant leaves, and local micro- and macrofauna (Lacerda et al. 2006 et al.; Paraquetti 2004; Fonseca et al. 2013; Victório et al. 2020; Souza et al. 2021; Silva et al. 2022a; Damasceno et al. 2024a, 2025). This region hosts large-scale industrial developments, including around 400 industries, the largest steelmaking complex in Latin America, and the Port of Itaguaí (Southeast), which is one of the country’s main iron ore export terminals (Lacerda et al. 2006).

Historically, family farming has supported the region's economy, serving as the main food supplier for the municipalities surrounding the study basin (SB), including schools, daycare centers, shelters, nursing homes, as well as the state capital (Freitas 1985; Corrêa 2016; Costa 2016; Prefeitura Municipal de Itaguaí 2017). Despite the agroecological relevance of the SB surroundings (Borges 2007) and the detection of PTE contamination in the SB and adjacent areas, there is no monitoring of food safety in the region.

In this context, this work aims to evaluate the concentrations of PTEs in agricultural products (roots, fruits, leaves, seeds, cheese and egg) cultivated and marketed by rural producers in the region surrounding Sepetiba Bay (SB, or SB region), one of Brazil's most contaminated coastal systems (Silva et al. 2022a). Also aiming to contribute to the advancement of global understanding of social vulnerability to environmental pollution by Potentially Toxic Elements. For this purpose, the results obtained in this work will be compared with levels established in regulations and with levels of PTEs recorded in other regions of Brazil and the world.

## Materials and Methods

### Study area

**Fig. 1:**
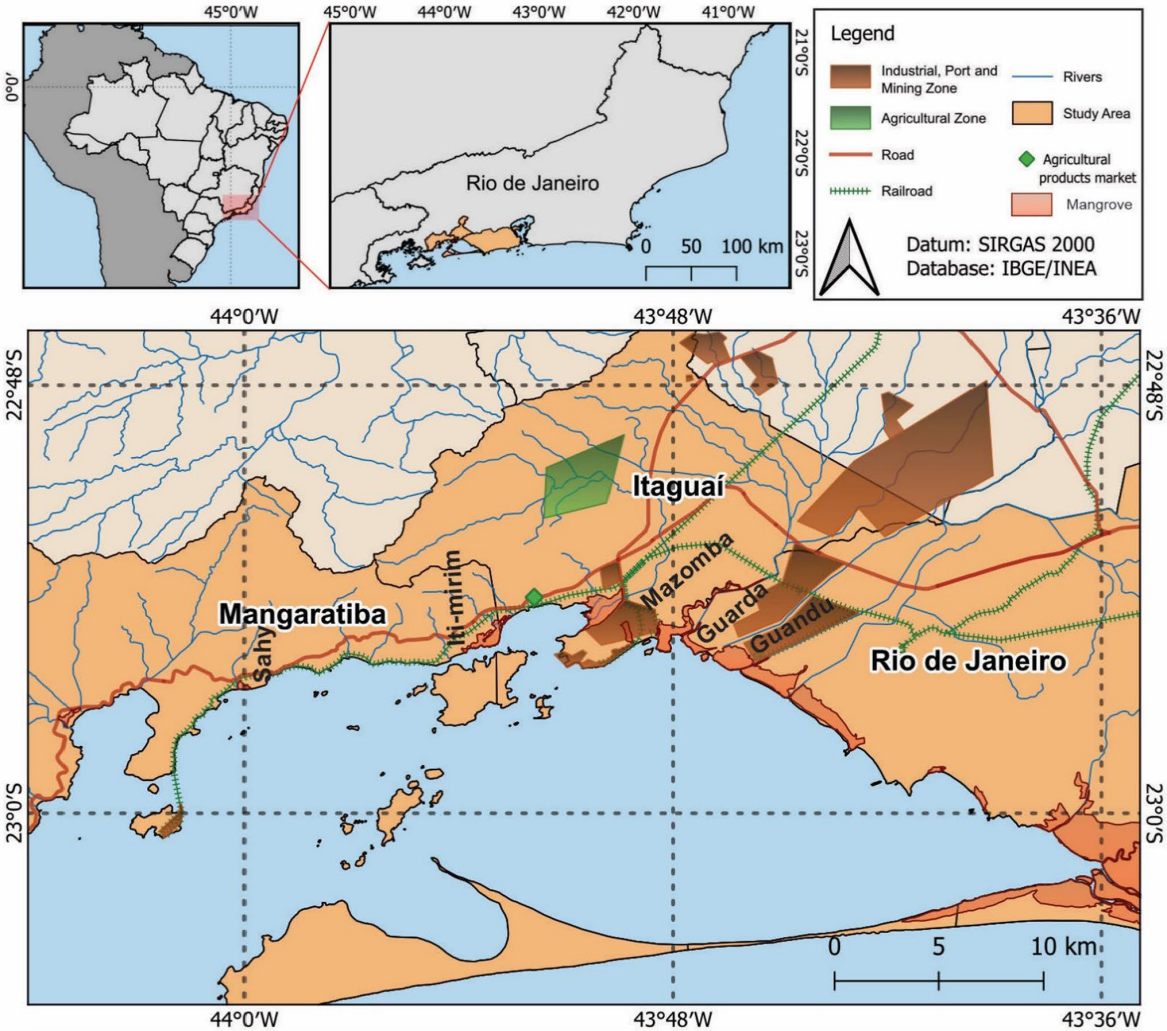
Location map of the study area. Based on data from IBGE (2023) and INEA (2023)

The study area encompasses rural zones surrounding Sepetiba Bay (SB) in Rio de Janeiro State, southeastern Brazil, within the metropolitan region of the capital, specifically in Itaguaí (Fig. 1). It is assumed that the activities occurring in this region may have an impact on these rural areas. To validate the importance of family farmers in the region surrounding Sepetiba Bay, cooperative practices were implemented to institutionally strengthen family-based agriculture through the Alimento Justo Program, within the framework of the Submarine Development Program (Marinha do Brasil 2016).

The SB has over 400 enterprises, among which port activities, ore transportation, fertilizers, steel and petrochemical industries stand out. Thus, the Port of Itaguaí handles approximately 45 million tons of iron ore exports, accounting for 87% of the total cargo moved through the port (ANTAQ 2014; PORTOSRIO 2021). Furthermore, the region is renowned for its scenic beauty and high volume of global tourism, primarily due to its proximity to the city of Rio de Janeiro (Leal Neto et al. 2006) and the Mangaratiba beaches.

The activities in the area underscore the geoeconomic and environmental significance of the Port of Itaguaí, as well as its direct influence on the socio-environmental dynamics and natural resources of the Sepetiba Bay surroundings. The region has high levels of pollution and enrichment of PTEs (Morales et al. 2019; Rodrigues et al. 2020; Souza et al. 2021; Castelo et al. 2021a,b; Silva et al. 2022a; Jeong et al. 2023; Saibro et al. 2023; Damasceno et al. 2024a).

### Soils in rural zones surrounding Sepetiba Bay (SB)

**Fig. 2:**
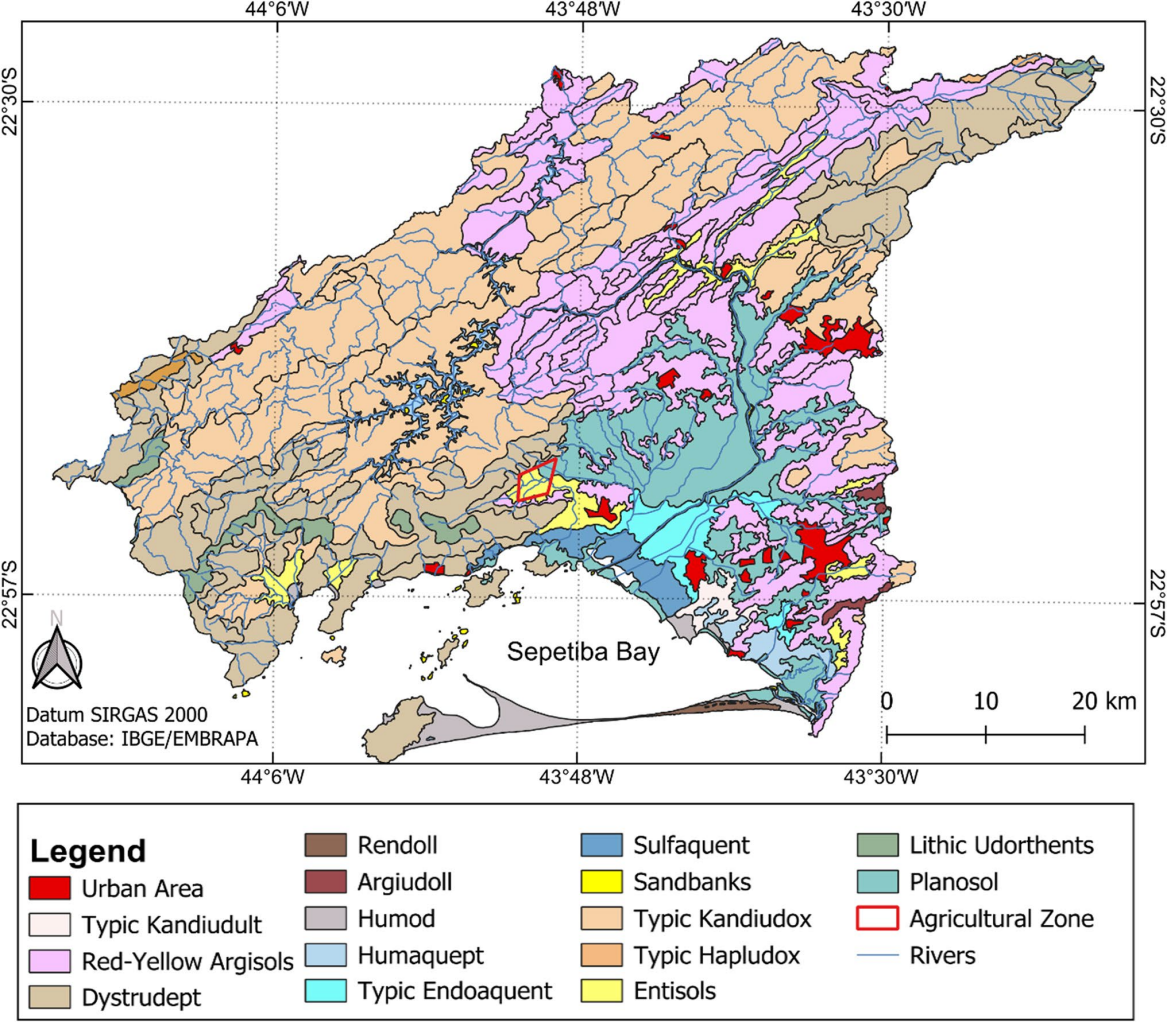
Map of the Soil Classification of the region surrounding Sepetiba Bay, based on IBGE, Embrapa and USDA Soil Taxonomy

Soil classification is relevant for investigating possible natural sources of PTEs in the region. The Soil Classification Map (Fig. 2) allows the visualization of the different soil classes of the municipalities surrounding SB. The foods selected for analysis in this study were primarily grown in neosols and, to a lesser extent, in planosols (Fig. 2).

Neosols are classified as young soils, typically located near their source rock, which results in the preservation of their original characteristics (Santos et al. 2018; Sacramento et al. 2019). The sedimentary framework can also be composed of alluvial grains (Santos et al. 2018). In this sense, the genesis of the sediments that comprise the neosols can vary in terms of color, texture, structure, and consistency (Silva and Silva 1997). Therefore, neosols are resistant to weathering (Santos et al. 2018). Consequently, it is common to find enrichment of certain metals in their chemical composition, originating from rocky outcrops (Santos et al. 2018).

The main characteristics of planosols are their ability to remove clay from the surface and accumulate it in the subsurface, resulting in a distinct layering (Macías and Camps-Arbestain 2020). These soils are formed through a pedochemical phenomenon called ferrolysis, which involves other processes such as oxidation–reduction, hydrolysis, acidolysis, and desilication, where the influence of groundwater controls chemical reactions (Barbiero et al. 2010).

Both neosols and planosols occur preferentially in reliefs ranging from flat to undulating. However, neosols occur in regions ranging from mountainous to planar and can come into contact with aquifers (IBGE 2015; Santos et al. 2018). The neosols result from the influence of both the geology and geomorphology of the local area, associated with the transition zone between the escarpments of the Serra do Mazomba mountain (Proterozoic rocks) and the alluvial sediments (Quaternary) around the Mazomba River (Fig. 1; Heilbron et al. 2016; Simão and Dantas 2021).

Planosols tend to occur in flat to mildly rugged regions and can be found in lowlands (Santos et al. 2018), as is the case in the study area (Simão and Dantas 2021). Both types of soils can occur in association with drainage zones (Santos et al. 2018). Texturally, neosols are composed of sandy horizons, whereas planosols are distinguished by the presence of a clay horizon (Santos et al. 2018). Even with limitations for agricultural use, both types of soils are utilized in family farming (Santos et al. 2018; Saraiva et al. 2021). Planosols, due to their flooding character, are widely used for rice cultivation (Santos et al. 2018).

The region surrounding SB, where the foods analyzed in this work were produced, is located in the lowland of Mazomba Mountain (Serra do Mar ridges). The sediments that comprise the region's soils are associated with the Coastal Deposits (Quaternary Deposits; Heilbron et al. 2016). These deposits are predominantly sandy and have been or are directly influenced by river and sea currents (Silva 2001).

In the mineralogical sense, the mountains of the region, in general, are composed of: quartz (SiO₂), feldspars [(Ca, Na)(Al, Si)₄O₈; (KAlSi₃O₈)], micas [K(Mg, Fe)₃(AlSi₃O₁₀)(OH)₂], mafic and accessory minerals (10–28%; André et al. 2018). The oldest rocks belong to the Juiz de Fora Complex, represented by gneisses of granitic origin (Araujo et al. 2021). The Ribeirão das Lajes formation consists of igneous lithotypes and high metamorphic grade, such as charnockites and granulites (André et al. 2018). The Rio Negro Complex is composed of the youngest gneisses in the region and features bands with high percentages of biotite and garnet (Tupinambá et al. 2000; Heilbron et al. 2020).

Recent studies on the mineralogy of sediments at the mouth of the Mazomba River, a water body that traverses the food production area (Fig. 1), have described the presence of minerals with high adsorption capacity, including kaolinite, biotite, and iron and aluminum oxides (Rodrigues et al., 2005). The Mazomba River is part of the Guandu River basin, one of the most important in the Rio de Janeiro state (Leal Neto et al. 2006). Furthermore, the geomorphological characteristics of the region surrounding Sepetiba Bay are typical of coastal plains, characterized by extensive mangrove forests (Fig. 1). These ecosystems are frequently flooded due to tidal dynamics (Menéndez et al. 2020).

### Food collection

The analyzed foods are cultivated by rural producers in the region of Itaguaí (22°53′56.65"S/ 43°51′52.87"W and 22°55′3.10"S/ 43°50′58.40"W). The main soils in the region where the foods were produced are neosols and, to a lesser extent, planosols (Fig. 2), as mentioned, naturally irrigated by the Mazomba River (Fig. 1).

Samples from 26 species were obtained and analyzed: *Capsicum annum* (peppers), *Solanurn lycopersicum* (tomato), *Allium cepa* (white onion), *Ananas comosus* (pineapple), *Musa acuminata* Colla (banana), *Daucus carota* (carrot), *Persea americana* (avocado), *Solanum melongena* (Eggplant), *Manihot esculenta* Crantz (cassava), *Ipomoea batatas* (sweet potato), *Solanum tuberosum* (potato), *Cucurbita moschata* (pumpkins), *Cucurbita maxima* (pumpkins), *Colocasia esculenta* (yam), *Arachis hypogaea* (peanut), *Capsicum chinense* (pout pepper), *Lactuca sativa* (lettuce), *Capsicum frutescens* (chili pepper), *Persea americana* (avocado), *Phaseolus vulgaris* (red, green and white beans), *Vigna unguiculata* (string beans, black-eyed peas), *Phaseolus lunatus* (fava beans), *Bos taurus* (cheese—cow) and *Gallus gallus domesticus* (egg—chicken).

The selection of products was intentional, prioritizing the foods most commonly consumed by residents of the region. However, the number of samples did not follow a systematic pattern. All food items were purchased in a single transaction on February 14, 2023, at one of the sales points of the family farmers' cooperative (Fig. 1). The products referred to were transported to the Micropaleontology Laboratory (LABMICRO) of the State University of Rio de Janeiro (UERJ). The samples were sanitized, sectioned, stored in plastic jars and frozen in a freezer. Subsequently, the samples were dehydrated in a freeze dryer (Martin Christ 1–2 Dlplus) for 60/72 h. After drying, the samples were macerated (reduced to powder) and submitted to elemental analysis. The geochemical analyses were conducted in the Inorganic Elements Sector (SELIN) of the Department of Chemistry at the National Institute for Control and Quality in Health (INCQS), located at the Oswaldo Cruz Foundation (FIOCRUZ), as described below.

### Chemical analysis

At the SELIN, approximately 0.3 g of each sample was weighed in duplicate and transferred to Teflon tubes for acid digestion. Then, 2 mL of deionized water (Millipore, Brazil), 2 mL of Suprapur nitric acid (65% (w/v); Merck, Germany), and 2 mL of hydrogen peroxide (30% (v/v); Merck, Germany) were added. The samples were digested for 1 h in a closed high-pressure system using the SpeedWave microwave (Berghof, Germany). After cooling, the samples were transferred to 15-ml Falcon tubes (Santos et al. 2023).

### Reagents and standards

Multielement Calibration Standard 3 (PerkinElmer) stock solution was used to prepare an intermediate solution of 1000 µg L⁻^1^, and then a second solution of 50 µg L⁻^1^ was prepared. From this intermediate solution (50 µg L⁻^1^), a calibration curve was prepared through successive dilutions, with a working range of 0.1–10 mg kg^−1^ for As, Cd, Pb, Cu, Zn, Ni, Co, Cr, and 0.05–2.5 mg kg^−1^ for Hg. To minimize potential interferences, a rhodium solution (Merck, Germany) with a final concentration of 10 mg kg^−1^ was added to the samples, standards, and blanks. The accuracy and precision of the method were evaluated using the National Institute of Standards and Technology (NIST Rice Flour 1568b) certified reference material and recovery studies. To assess recovery, known quantities of each analyte were added to peanut and onion samples at a concentration level of 5 mg kg^−1^. These studies were conducted to ensure the reliability of the results (INMETRO 2020; ISO 2017a,b).

### Instrumentation

The concentrations of the chemical elements (As, Cd, Pb, Cu, Zn, Ni, Co, Cr, and Hg) were determined with inductively coupled plasma mass spectrometry (ICP-MS) model NexION 300D, PerkinElmer, USA) equipped with a concentric nebulizer (MEINHARD® Plus), glass cyclonic nebulizer chamber, cone, skimmer and nickel hyper-skimmer. Argon gas, with a minimum purity of 99.996%, was provided by White Martins (São Paulo, Brazil).

Several legal documents were used to analyze the chemical elements’ concentration range in the analyzed foods. The Brazilian legislation ANVISA (Normative Instruction—IN Nº. 88, of March 26, 2021, and Ordinance Nº. 685, August 27, 1998) defines the values for As (from 0.3 mg kg^−1^, 0.1 mg kg^−1^ and 0.5 mg kg^−1^ for fruits, vegetables and eggs, respectively), Cd (0.05 mg kg^−1^) and Pb (from 0.1 mg kg^−1^, 0.2 mg kg^−1^ and 0.4 mg kg^−1^, for vegetables, beans, and cheese, respectively) and Cu (10 mg kg^−1^). ANVISA (2021) does not specify limits for Ni, Zn, Cr, and Hg. Therefore, the standards released by the United States Department of Agriculture—Foreign Agricultural Service carried out by the China team (USDA-FAS 2023) for Ni (1.0 mg kg^−1^), Cr (0.5 mg kg^−1^- vegetable and vegetable products; 2.0 mg kg^−1^—fermented milk) and Hg (0.01 mg kg^−1^) and ANVISA (1965) for Zn (60 mg kg^−1^), were used in this work.

### Validation

The validation of the analytical method was carried out in accordance with the document “Validation of Analytical Methods” by the National Institute of Metrology, Quality and Technology—INMETRO (2020) and ISO (2017a,b).

The determination of the limit of detection (LOD) was performed through the reading of 10 independent solutions from the blank, with calculations according to INMETRO (2020) guidelines, ensuring 95% reliability. The LOD was experimentally defined as the first point of the calibration curve (INMETRO 2020).

To assess the method's accuracy and precision, certified reference materials NIST 1568b were used, following acceptance criteria established by INMETRO (2020), which allow a range between 60 and 120% of the certified value, with a maximum relative standard deviation (% RSD) of less than 20% (INMETRO 2020; ISO 2017a,b).

The maximum permissible limits for potentially toxic elements (PTEs) in food were established according to the regulations of the following agencies: National Health Surveillance Agency (ANVISA 2021), World Health Organization/Food and Agriculture Organization (WHO/FAO 2024), and the United States Department of Agriculture (USDA-FAS 2023). The working range adopted varied according to the Collegiate Board of Directors Resolution—RDC No. 42, of August 29, 2013, regarding the Southern Common Market (MERCOSUR) (Table 1).

**Table 1:** Parameters for determination and quantification of metals by ICP-MS

Instrumental parameters	
RF power 1400W	
Argon gas flow rate	
Plasma 17 L min^−1^	
Auxiliary 1.1 L min^−1^	
Nebulizer 1 L min^−1^	
Scanning mode peak hoping	
Point per spectral peak 1	
Sweeps 20	
Dwell time 50 ms	
Readings per replicate 1	
Settle time 3	
Integration time 1 s	
Internal standard Rh (10 μg L^−1^)	

Control and precautionary measures were ensured through the use of certified analytical reagents, blank preparation, replication (in duplicate) of digestion procedures, and the use of reference materials 1568b—IAEA-359 (ISO 2017a; Supplementary Table 1), ensuring the quality and reliability of the analyses.

### Statistical analysis applied to the concentrations of PTEs in the studied foods

Descriptive statistics were performed using Microsoft Excel 2010, where the arithmetic mean (duplicate) and standard deviation (SD), Student's t-test, and analysis of variance (ANOVA) were calculated for metals.

Before the statistical analyses, the PTE concentrations found in the foods were transformed using the log (*x* + 1) function. Spearman rank order correlations were used to evaluate the correlations between the variables. Correlations were considered significant for *p* < 0.05. Cluster analyses (using the Ward method and 1-Pearson r for grouping) and principal component analysis (PCA) were employed to investigate the variability of chemical elements in the studied foods. Statistical analyses were performed using STATISTICA 12 software (StatSoft) and maps using QGIS software.

### Food contamination risk assessment

To evaluate the probability of food risk to human health in relation to metal enrichment, the Estimated Daily Intake (EDI), Hazard Quotient (HQ), and Hazard Index (HI; non-carcinogenic risk) were calculated. The potential risk to consumer health caused by PTE, as measured by the EDI (mg/day) in adults through daily vegetable intake (DI), was calculated using Eq. (1) (Rattan et al. 2005; Augustsson et al. 2018).

1 $$ {\text{EDI}}\, = \,{\text{CP}}*{\text{Cf}}*Df\omega t $$

where CP is the concentration of the element in the sample (mg kg^−1^; dw), Cf or Cfactor is the conversion factor from dry to fresh weight (0.086; Rattan et al. 2005), Df or Dfood is the fresh vegetables per person (0.40 kg day^−1^); where wt is the average total body weight (70 kg for adults; Ogunkunle et al. 2015, 2016, 2022; Edogbo et al. 2020).

The non-carcinogenic risk of PTEs from the EDI of individual plants to residents was calculated using the Hazard Quotient (HQ), based on Eq. (2) (Ogunkunle et al. 2022).

2 $$ {\text{HQ}}\, = \,{\text{EDI}}/{\text{RfD}} $$

where RfD is the universal Reference Dose (RfD) for all metals but provides guidelines for specific ones (FAO/WHO 1995–2023, Expert Committee on Food Additives; Table 2).

**Table 2:** Daily reference intake (Rfd) of PTEs (mg kg^−1^ of body weight per day; Adhikar et al. 2022; Sikakwe et al. 2024)

PTEs		Rfd
As	mg kg^−1^	0.0003
Cd	mg kg^−1^	0.001
Co	mg kg^−1^	0.03
Cr	mg kg^−1^	0.003
Cu	mg kg^−1^	0.04
Hg	mg kg^−1^	0.005
Ni	mg kg^−1^	0.02
Pb	mg kg^−1^	0.0035
Zn	mg kg^−1^	0.3

The Hazard Index (HI) suggests potential consequences of PTEs in humans. HI was calculated as the sum of all HQs (Eq. 3; Ametepey et al. 2018; Ogunkunle et al. 2022).

3 $$ {\text{HIPTES}}\, = \,i\, = \,{1}n{\text{HQ}} $$

When the HI value is less than 1, it suggests no dietary risk to humans, while a value above 1 implies a potential risk associated with consuming the analyzed foods (Kormoker et al. 2020; Ogunkunle et al. 2022).

## Results

The limit of quantification, defined as the first point of the calibration curve, was 0.1 mg kg-1 for As, Cd, Pb, Cu, Zn, Ni, Co, and Cr, and 0.05 mg kg-1 for Hg. Regarding the recovery assessment, Cd, Pb, and Zn showed values above the acceptable range of 60–120%. However, this variation does not affect the results, indicating that the methodology is suitable for the study. For the other elements, the recoveries were within the acceptance criteria. The recovery values for NIST 1568b were consistent with the accepted range of 60–120%; however, the sample does not contain Pb, Co, or Hg. The precision, evaluated by the relative standard deviation (% RSD), was below 20% for all the studied elements. Thus, the optimized and validated methodology presented in this study proved to be suitable for determining various inorganic elements in environmental samples using Inductively Coupled Plasma Mass Spectrometry (ICP-MS).

### Concentrations of PTEs in agricultural products

Concentrations of Zn, Cu, Ni, and Cr were measured in all the foods (Appendix 1 in ESM; Table 3; Supplementary Fig. 1; Fig. S1). The elements that reached the highest concentrations in the analyzed foods were Zn (range: 0.60–143.8 mg kg^−1^; mean or x̅ = 27.0 ± 25.46 mg kg^−1^) and Cu (range 0.57–105.3 mg kg^−1^; x̅ = 16.4 ± 20.75 mg kg^−1^). Some elements reached maximum concentrations between 2–10 mg kg^−1^: Ni (range 0.043–9.580 mg kg^−1^; x̅ = 1.752 ± 2.230 mg kg^−1^), Cr (range 0.190–7.814 mg kg^−1^; x̅ = 2.294 ± 2.292 mg kg^−1^) and As (< 2.083 mg kg^−1^; x̅ = 0.205 ± 0.434 mg kg^−1^). Lead concentrations reached 0.814 mg kg^−1^ (x̅ = 0.16 ± 0.22 mg kg^−1^). Those of the other chemical elements were < 0.5 mg kg^−1^ in the analyzed foods: Co (0.001–0.378 mg kg^−1^; x̅ = 0.081 ± 0.093 mg kg^−1^), Cd (< 0.093 mg kg^−1^; x̅ = 0.019 ± 0.024 mg kg^−1^) and Hg (< 0.087 mg kg^−1^; x̅ = 0.021 ± 0.025 mg kg^−1^).

**Table 3:** Metal ranges of concentrations in the analyzed foods. Limits stipulated by legislation ANVISA (Normative Instruction—IN Nº. 88, of March 26, 2021, and Ordinance No. 685, of August 27, 1998); all values in (mg kg^−1^)

Metals		Maximum	Minimum	Mean	SD	Limits	
Zn	(mg kg^−1^)	143.82	0.60	27.01	25.46	60	ANVISA 1965
Cu	(mg kg^−1^)	105.28	0.57	16.45	20.75	10	ANVISA 2021
Ni	(mg kg^−1^)	9.58	0.04	1.75	2.23	1.0	USDA-FAS 2023
Cr	(mg kg^−1^)	7.81	0.19	2.29	2.29	0.5—vegetable and vegetable products, 2.0 fermented milk	USDA-FAS 2023
As	(mg kg^−1^)	2.08	0.00	0.21	0.43	0.3—fruits, 0.1—vegetables, 0.5—eggs	ANVISA 2021
Pb	(mg kg^−1^)	0.81	0.00	0.16	0.22	0.1—vegetables, 0.2—beans, 0.4—cheese	ANVISA 2021
Co	(mg kg^−1^)	0.38	0.00	0.08	0.09	–-	–-
Cd	(mg kg^−1^)	0.09	0.00	0.02	0.02	0.05	ANVISA 2021
Hg	(mg kg^−1^)	0.09	0.00	0.02	0.02	0.01	USDA-FAS 2023

**Fig. 3:**
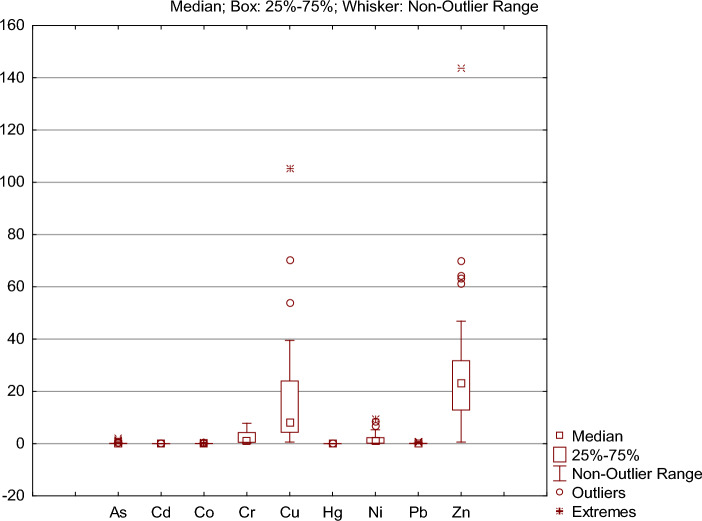
Ranges of the PTE concentrations in the analyzed foods. The non-outlier range, median, variability of 25–75% of the data and extreme values are presented

The boxplots in Fig. 3 present the non-outlier range, median, variability between the 25th and 75th percentiles, and extreme elemental concentrations in the analyzed foods. The elements with the highest median and range values between 25–75% were Zn and Cu; these elements also exhibited the highest values of outliers and extreme concentrations (Fig. 3A). Nickel reached relatively high outlier and extreme concentrations in the analyzed foods (Fig. 3B, C). Arsenic presented a high number of outliers and extreme values, standing out compared to Pb and Co (Fig. 3C). Mercury and Cd elements were more homogeneous, with small ranges and few outliers (Fig. 3C).

**Fig. 4:**
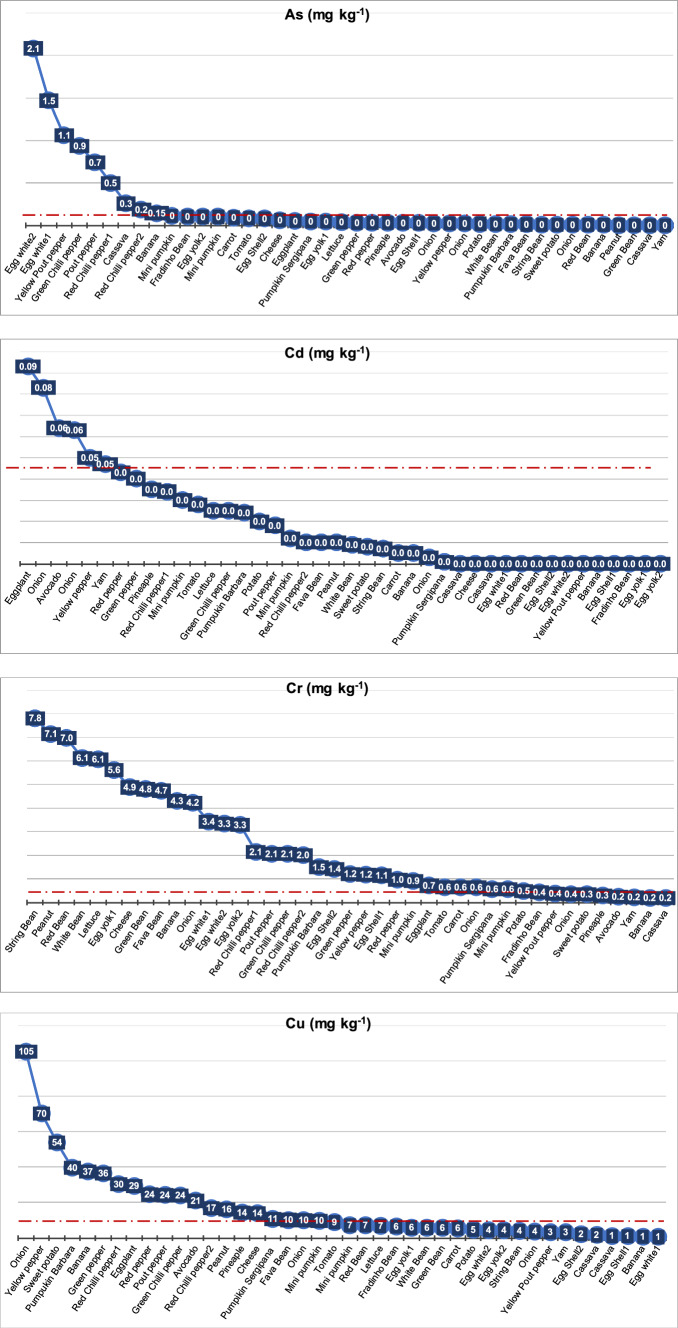

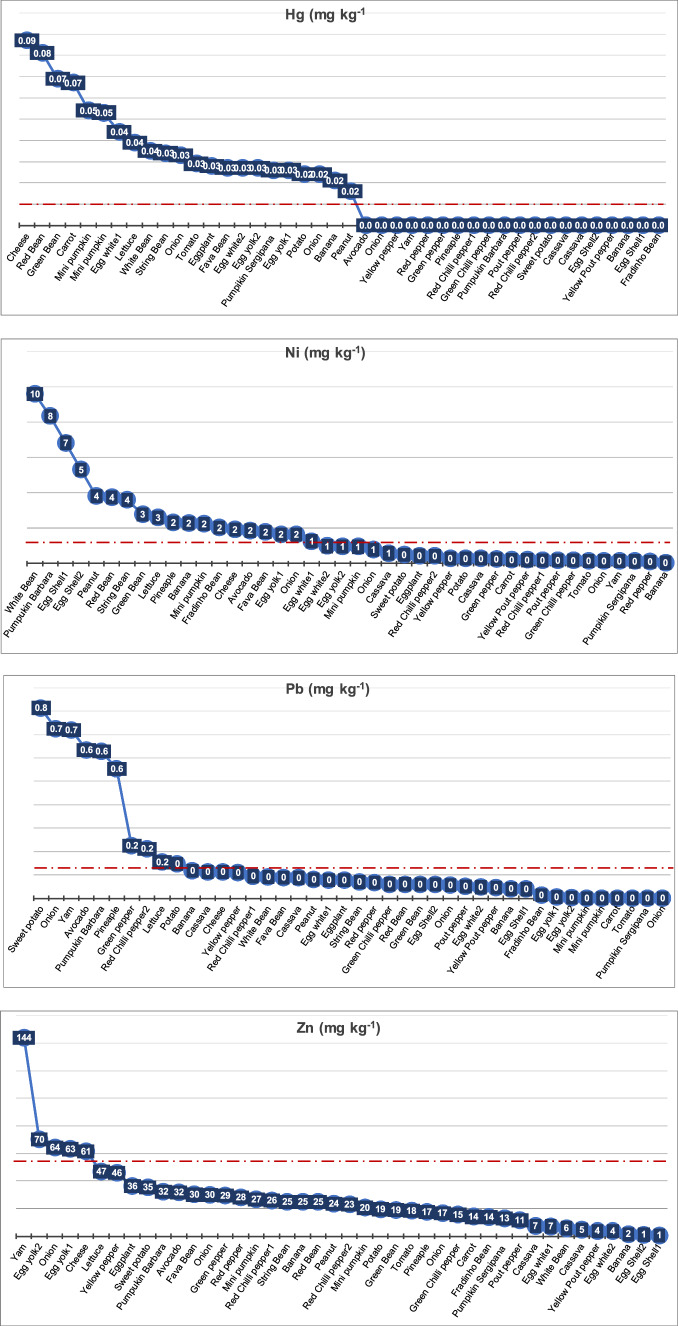
The PTE concentrations (mg kg^−1^) in the analyzed food items. The dashed red line represents limits stipulated by regulatory authorities, according to Table 3

The bar plot in Supplementary Fig. 2 (Fig. S2) shows the concentration of metals in each food. The graphs in Fig. 4 (Appendix 1 in ESM ) show that several foods reached concentrations higher than those indicated by regulatory entities (ANVISA 1965, 2021; USDA-FAS 2023; see Table 3). The heat map was designed to facilitate the visualization of foods that exceed the limits established by regulatory agencies, the values that are relatively high and the levels that present a higher degree of safety (Fig. S2) for:

- **Zn** (> 60 mg kg^−1^)—yam (*C. esculenta),* egg yolk (from *G. gallus domesticus),* white onion (*A. cepa),* and cheese (of cow's milk; *B. taurus*);

- **Cu** (> 10 mg kg^−1^) —onion (*A. cepa),* yellow pepper (*C. annum),* sweet potato (*I. batatas),* pumpkin barbara (*C. moschata*), banana (*M. acuminata Colla*), green pepper (*C. annum),* red chili pepper (*C. frutescens*), Eggplant (*S. melongena*), pout pepper (*C. chinense*), green chili pepper (*C. chinense*)*,* avocado (*P. americana),* peanut (*A. hypogaea*), pineapple (*A. comosus*), cheese (made with cow's milk; *Bos taurus*), and pumpkin sergipana (*C. maxima*)*.*

- **Ni** (> 1.0 mg kg^−1^) —white, green and red bean (*P. vulgaris*), Pumpkin barbara (*C. moschata*)*,* eggshell, yolk and white (*G. gallus domesticus*)*,* peanut (*A. hypogaea),* string and “Fradinho” bean (in Brazil) or cowpea (*V. unguiculata*)*,* lettuce (*L. sativa*)*,* Pineapple (*A. comosus*)*,* banana (*M. acuminata*), cheese (of *B. taurus*)*,* avocado (*P. americana*), fava bean (*P. lunatus*), and onion (*A. cepa*)*.*

- **Cr** (0.5 mg kg^−1^—vegetable and vegetable products, 2.0 mg kg^−1^ fermented milk)—most of the analyzed food items except potato (*S. tuberosum),* cowpea (*V. unguiculata),* yellow pout pepper (*C. chinense),* onion (*A. cepa),* sweet potato (*I. batatas),* pineapple (*A. comosus),* avocado (*P. americana),* yam (*C. esculenta),* banana (*M. acuminata),* and cassava (*M. esculenta).*

- **As** (0.3 mg kg^−1^—fruits, 0.1 mg kg^−1^—vegetables, 0.5 mg kg^−1^– eggs) —egg white (*G. gallus domesticus*), pout pepper *(C. chinense*), green chili pepper (*C. frutescens),* cassava (*M. esculenta),* banana (*M. acuminata*), and mini pumpkin (*C. moschata).*

- **Pb** (> 0.1–0.4 mg kg^−1^)—onion (*A. cepa),* yam (*C. esculenta),* avocado (*P. americana),* pineapple (*A. comosus*), green pepper (*C. annum),* red chili pepper (*C. frutescens),* lettuce (*L. sativa).*

- **Cd** (0.05 mg kg^−1^)—eggplant (*S. melongena*), onion (*A. cepa),* avocado (*P. americana),* yellow pepper (Y. pepper), and Yam (*C. esculenta).*

- **Hg** (0.01 mg kg^−1^)—cheese (of cow's milk; *B. taurus*); red, green, white, and string beans (*P. vulgaris, S. bean),* carrot (*D. carota*), mini pumpkin (*C. moschata),* egg white (*G. gallus domesticus),* lettuce (*L. sativa),* onion (*A. cepa),* eggplant (*S. melongena*), fava bean (*P. lunatus),* egg white and yolk (*G. gallus domesticus),* pumpkin sergipana (*C. maxima),* potato (*S. tuberosum),* banana (*M. acuminata),* and peanut (*A. hypogaea*).

### Statistical results

**Fig. 5:**
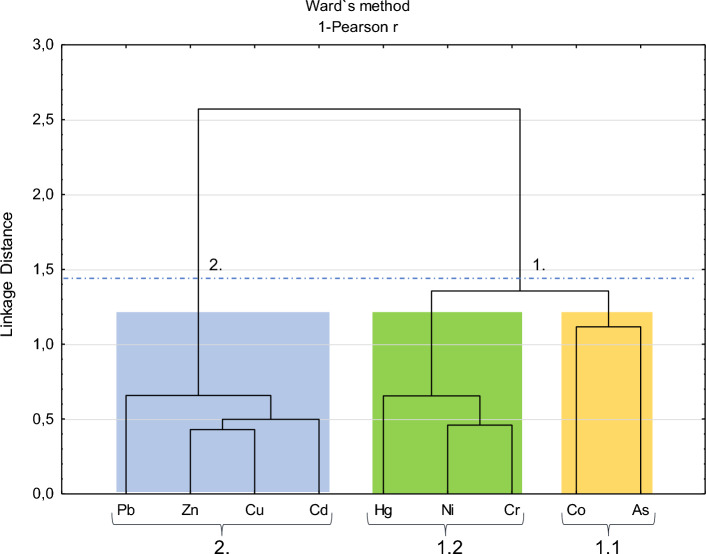
Cluster analysis (CA) based on the elemental concentrations (R-Mode) in the analyzed food items. The main clusters and sub-clusters of the elements were identified

The cluster analysis (CA), based on the elemental concentrations in the analyzed food items, identified two main groups of variables (Fig. 5). In group 1, two subgroups can be distinguished: Subgroup 1.1, which includes Co and As, and Subgroup 1.2, which encompasses Hg, Ni, and Cr. Group 2 includes Pb, Zn, Cu, and Cd (Fig. 5). These groups/subgroups have chemical elements with similar distribution trends in the analyzed foodstuffs, as indicated by the correlations presented in Appendix 2 in ESM.

**Fig. 6:**
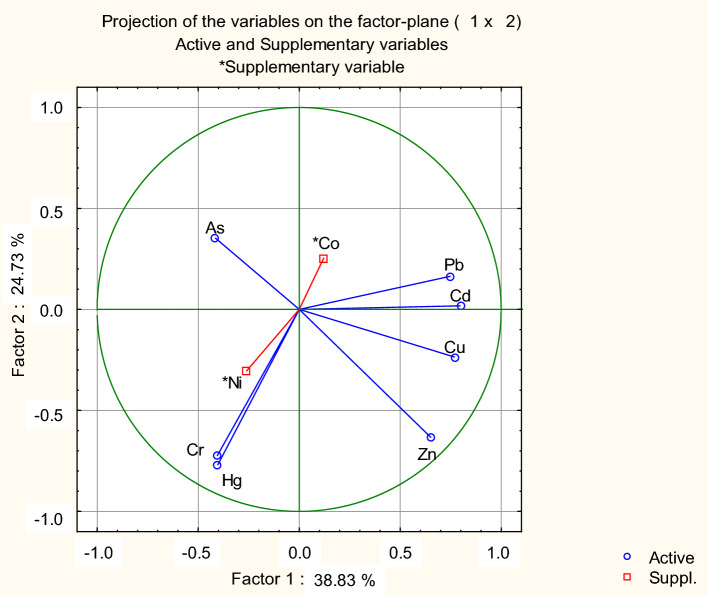
Principal component analysis (PCA) based on the concentrations of the chemical elements in the analyzed food items. Cobalt and Ni were used as supplementary variables (Suppl.) since the food either reaches concentrations below those recommended (in the case of Ni) or the risk limit is not known (in the case of Co). The remaining elements were active variables

The distribution of the variables in the first two factors of the principal component analysis (PCA; Fig. 6) is similar to that of the CA (Fig. 5). These first two factors of the PCA explain most of the data variability (63.56%; Factor 1: 38.83%; Factor 2: 24.73%), which gives the reliability to the groups established by the CA (Fig. 6).

**Fig. 7:**
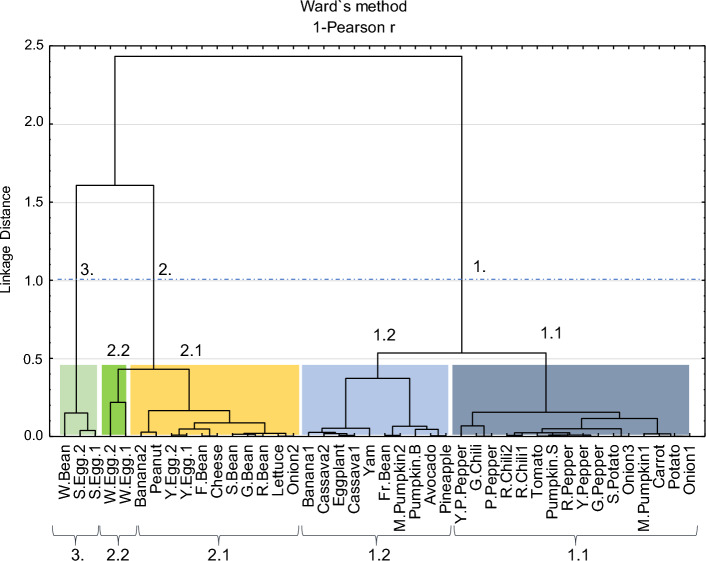
Cluster analysis groups (Q-Mode) based on the elemental concentrations of the analyzed food items. The main clusters and sub-clusters of food items were identified

The CA presented in Fig. 7 groups the analyzed food items, based on their elemental concentrations, into three main groups. In groups 1 and 2, two subgroups were identified. The correlations presented in Appendix 2 in ESM justify the establishment of these groups and subgroups, as well as the following levels of average metal concentrations:

Subgroup 1.1: with the highest concentrations of Zn and Cu and Cd; the average concentrations of Cu and Cr are higher than the limits set by the authorities (ANVISA 2021);

Subgroup 1.2: with the highest concentrations of Pb, and relatively high Zn and Cu contents and low levels of Cr; the average concentrations of Pb and Ni are higher than the limits set by the authorities (ANVISA 2021);

Subgroup 2.1: with the highest concentrations of As and relatively high levels of Hg and Cr, lower levels of Pb, Cu, Ni, Co; Cd were not detected; the average concentrations of Cr and Hg are higher than the limits set by the authorities (USDA-FAS 2023);

Subgroup 2.2: with the highest concentrations of Cr and Hg, high Zn contents, relatively high concentrations of Ni and intermediate contents of As and Cu, the average concentrations of As, Ni, Cr and Hg are higher than the limits set by the authorities (ANVISA 2021; USDA-FAS 2023);

Group 3: with the highest concentrations of Ni and Co, and relatively high contents of Cr, average concentrations of Ni and Cr are higher than those stipulated by the authorities (USDA-FAS 2023); low contents of As, Cd, Cu, Zn.

Indices of dietary intake probability and risk to human health were calculated (Appendix 3 in ESM; Table 4). EDI values ranged from 0 to 0.07, while calculated HQ levels ranged from 0 to 2.87. The most significant HQ values were associated with the presence of As and Cr, particularly in the following species: *P. vulgaris* (red, green and white beans), *G. gallus domesticus* (eggshell, yolk and white), *B. taurus* (cheese), *V. unguiculata* (cowpea), *P. lunatus* (fava bean), *L. sativa* (lettuce), and *A. cepa* (Onion). In this context, the HI values calculated from HQ were high (> 1) in descending order in the following food items: *G. gallus domesticus* eggs (white egg > eggshell > yolk egg), *C. chinense* (pout pepper), A*. hypogaea* (peanut), *L. sativa* (lettuce), P*. vulgaris* (red bean, white bean), cheese of *B. taurus*, and *V. unguiculata* (string bean).

**Table 4:** Possible risks of analyzed PTEs on human health

Element	Toxicity and Health Effects	Associated Diseases/Conditions	Carcinogenic Classification (IARC)
Zn (Zinc)	Essential in small amounts; excess can cause nausea, vomiting, immune alterations, and anemia	Gastrointestinal toxicity, hypocupremia (copper deficiency), metabolic alterations	Not classified as carcinogenic
Cu (Copper)	Essential, but excess may cause oxidative stress, liver and kidney damage	Wilson's disease (chronic accumulation), hepatotoxicity, nephrotoxicity	Not classified as carcinogenic
Ni (Nickel)	Immunotoxic and genotoxic effects; skin sensitizer	Allergic dermatitis, lung and paranasal sinus cancer (in occupational exposure)	Group 1—Carcinogenic to humans
Cr (Chromium)	Chromium (VI) is highly toxic: genotoxic, immunotoxic, and carcinogenic	Lung cancer, dermatitis, skin ulcers, occupational asthma	Group 1—Carcinogenic (for Cr VI)
As (Arsenic)	Highly toxic; chronic effects include skin changes and cancer	Skin, lung, and bladder cancer, cardiovascular diseases, neurotoxicity, type 2 diabetes	Group 1—Carcinogenic to humans
Pb (Lead)	Neurotoxic; affects central nervous system, especially in children	Cognitive deficits, behavioral changes, nephrotoxicity, hypertension, anemia	Probably carcinogenic (Group 2A)
Co (Cobalt)	Essential in small amounts (vitamin B12), but excess can be toxic	Pulmonary fibrosis, cardiomyopathy, cancer (in occupational exposure)	Group 2B—Possibly carcinogenic
Hg (Mercury)	Potent neurotoxin; bioaccumulates in the food chain	Minamata syndrome (neuropathy), renal and immune alterations, fetal developmental effects	Not classified as carcinogenic but highly toxic
Cd (Cadmium)	Highly toxic and bioaccumulative; genotoxic	Lung, prostate, and kidney cancer, chronic kidney disease, osteoporosis, Itai-Itai syndrome	Group 1—Carcinogenic to humans

Source: Adapted from ATSDR (2019–2022), IARC (2022–2023), WHO (2021), Plum et al. (2010), Gaetke and Chow (2003)

## Discussion

### Comparison with the limits referred to by the regulatory bodies

In some foods, As, Cd, Pb, Cu, Zn, Cr, and Hg concentrations exceed the limits defined by regulatory agencies (ANVISA 1965, 2021; USDA-FAS /China 2023; Appendix 1 in ESM and Table 3; Fig. S2). Concentrations above the limits established by law were detected in: *A. cepa* (onion) for Cd, Pb, Cu, Ni, Cr, and Hg; *A. comosus* (pineapple) for Cu, Ni, Cr, and Hg; *B. taurus* cheese for Cu, Zn, Ni, Cr, and Hg; *C. annum* (green, red, yellow pepper) for Cu, Cr, and Hg; *C. chinense* (pout and yellow pout pepper) for As, Cu, and Cr; *C. frutescens* (green and red pepper) for As, Cu, and Ni; *C. esculenta* (Yam) for Pb and Zn; *C. maxima* (pumpkin sergipana): Cu, Cr, and Hg; *C. moschata* (mini pumpkin and pumpkin barbara) for Pb, Cu, Ni, Cr, and Hg; *D. carota* (carrot) for Cr and Hg *G. gallus domesticus* (chicken)—egg yolk for Zn, Ni, Cr, and Hg, egg white for As, Ni, and Cr and eggshell for Ni and Cr; *I. batatas* (sweet potato) for Pb and Cu; *L. sativa* (lettuce) for Ni, Cr, and Hg; *M. esculenta* (cassava) for As; *M. acuminata* (banana) for Ni, Cr, and Hg; *P. americana* (avocado) for Cd, Pb, Cu, and Ni; *P. lunatus* (fava beans) for Ni, Cr, and Hg; *P. vulgaris* (red, green and white beans) for Ni, Cr, and Hg; *S. lycopersicum* (tomato) for: Cr, and Hg; *S. melongena* (eggplant) for Cd, Cu, Cr, and Hg; *S. tuberosum* (potato) for: Hg and; *V. unguiculata* (string beans and cowpeas) for Ni, Cr, and Hg. It should be noted that in various specimens of the same species, the enrichment in metals was not always identical (Appendix 1 in ESM); this could be related to cultivation in areas with different characteristics and varying degrees of contamination, as well as ontogenetic effects, among other factors.

The three samples of *A. cepa* (onion 1, 2, 3) presented significant concentrations of the analyzed PTEs. The maximum values of Cu, Cr, Pb, Hg, Cd and Zn in *A. cepa* (onion; Appendix 1 in ESM) exceeded the levels stipulated by ANVISA (2021) by 10.5, 8.4, 7.2, 3.3, 1.6 and 1.0 times, respectively. The species *A. comosus* (pineapple) also exceeded the levels ruled by the regulatory agencies by 5.5 times for Pb and 1.4 times for Cu.

In *A. hypogaea* (peanut), Cu (14.3 times), Cr (1.6 times), and Hg (1.6 times) reached concentrations higher than the values defined by ANVISA (2021) and USDA-FAS (2023). Most of the studied elements were detected in cheese from *B. taurus*, where the maximum levels of Cr, Hg, Cu, and Zn exceeded the values admitted by ANVISA (2021) and USDA-FAS (2023) by 9.7, 8.7, 1.3, and 1.0 times, respectively.

In *C. annum* (green, red, and yellow pepper), the maximum values of Cu and Cr exceeded the values set by 7.0 and 2.4 times, respectively. Meanwhile, the maximum values of Cd and Pb are slightly above the limits allowed by law, as per ANVISA (2021) and USDA-FAS (2023). The maximum limits of Cu and Cr exceed 4.1 and 2.4 times the limit allowed by ANVISA (2021) in specimens of the same family, *C. chinense* (pout and yellow pepper). In addition, the two analyzed samples showed As levels 3.5 and 2.3 times higher, respectively, than the levels stipulated by ANVISA (2021).

Similarly, concentrations were found in the three analyzed samples of *C. frutescens* (green and red chili peppers 1 and 2), with a maximum concentration 3.1 times higher than the values defined by ANVISA (2021). The maximum levels of Cr, Cu, and Pb in these food items exceeded the limits specified by ANVISA (2021) and USDA-FAS (2023) by 4.0, 3.0, and 2.0 times, respectively. In *C. esculenta* (yam), significant Cd, Pb, and Zn contents were detected. The maximum concentrations of Pb and Zn were 7.2 and 2.4 times higher, respectively, than the values defined by ANVISA (2021).

*Cucurbita maxima* (pumpkin barbara 1, 2) revealed relatively high concentrations of Cu, Cr, and Hg. In this food item, the maximum Hg concentration was 2.6 times higher than the limit stipulated by USDA-FAS (2023). The species *C. moschata* (mini pumpkin 1, 2) showed enrichment of Cu, Cr and Hg similar to *C. maxima* (pumpkin barbara 1, 2)*.* However, the maximum concentrations of Cu, Cr, Hg and Pb were even higher and exceeded by 4.0, 3.0, 5.4, and 6.2 times, respectively, the values stipulated by the legislation. In *D. carota* (carrot), Cr and Hg exceeded the limits defined by USDA-FAS (2023) by 1.3 and 6.7 times, respectively.

Chromium and Hg also exceeded the values tolerated by USDA-FAS (2023) in eggs (from *G. gallus domesticus*). In yolk eggs (1, 2), the maximum Cr concentration was 11.2 times higher than the values allowed by the USDA-FAS (2023). Meanwhile, the maximum values of Hg and Pb were significantly higher (2.7 and 1.16 times, respectively) than those established by ANVISA (2021) and USDA-FAS (2023). The egg white (1, 2) exhibited maximum Cr, Hg, and As concentrations that were higher than those reported by ANVISA (2021) and USDA-FAS (2023), at 6.8, 4.4, and 4.2 times the respective levels. In the eggshell (1, 2), only Cr showed maximum concentrations higher than those considered by USDA-FAS (2023).

Concentrations of Pb and Cu were detected in *I. potatoes* (sweet potato) that were 8.1 and 5.3 times, respectively, higher than that stipulated by ANVISA (2021). All the analyzed PTEs were measured in *L. sativa* (lettuce); Cr and Hg exceeded the values defined by USDA-FAS (2023) by 12.1 and 4 times, respectively. *M. esculenta* (cassava) exhibited concentrations of As and Pb that were higher than the allowed values by 2.5 and 1.1 times, respectively (ANVISA 2021).

The maximum concentrations of Pb, Cu, Cr, and Hg in *M. acuminata* (banana 1, 2) also exceeded the limits accepted by ANVISA (2021) and USDA-FAS (2023) by 1.2, 3.7, 8.6, and 2.1 times, respectively. In *P. americana* (avocado), Cd, Cu, and Pb concentrations were higher than the values decreed by ANVISA (2021) at 1.3, 6.3, and 2.0 times, respectively.

In *P. lunatus* (fava beans), the concentrations of Cu, Cr, and Hg exceeded the limits set by ANVISA (2021) and USDA-FAS (2023) by 1.0, 9.4, and 2.7 times, respectively. The species *P. vulgaris* (red, green and white beans) also showed significant values of these PTEs. The maximum levels of Cr and Hg detected in *P. vulgaris* exceeded the limits defined by USDA-FAS (2023) by 14.0 and 7.0 times, respectively. The maximum Ni concentration in this species was the most significant among the investigated foods.

In *S. lycopersicum* (tomato), the Cr and Hg concentrations exceeded the limits considered by USDA-FAS (2023) by 1.3 and 3.0 times, respectively, and in *S. melongena* (eggplant), Cd, Cu, Cr, and Hg concentrations exceeded the limits set by ANVISA (2021), and USDA-FAS (2023) at 1.8, 3.0, 1.4 and 2.8 times, respectively. The samples of *S. tuberosum* (potato) showed Cr values close to those accepted by USDA-FAS (2023). However, Pb and Hg concentrations were 2.4 and 1.5 times higher, respectively, than the values stipulated by the ANVISA (2021) and USDA-FAS (2023).

In *V. unguiculata* (string and cowpea), As slightly exceeded the levels allowed by ANVISA (2021). However, Cr and Hg were 15.6 and 3.4 times higher, respectively, than the concentrations admitted by USDA-FAS (2023).

Copper, Zn, Ni, Co, and Cr were detected in all analyzed food items (Appendix 1 in ESM). In general, all foods had at least two of the elements detected above the limits defined by ANVISA (2021) and USDA-FAS (2023). Chromium concentrations above the levels stipulated by USDA-FAS (2023) were observed in more than 75% of the analyzed food items. In all foods, Hg concentrations were > 0.005 mg kg^−1^, exceeding the limits established by the USDA-FAS (2023; Appendix 1 in ESM).

### Comparison with other places in Brazil and the world

The mean concentrations in *A. cepa* (onion) produced in SB region (data of this work; Appendix 4 in ESM) for: As, Pb, Cu, Zn, Cr, and Hg were higher than that in Liaoning Province, a region of intensive industrial activity in China (Li et al. 2016); Zn was higher compared to specimens collected in the Kosovo Thermoelectric Power Plants (Zeneli et al. 2013); Cd, Pb, and Cr were higher than the mean levels found in this species in the Siran Valley (Pakistan; Tahir et al. 2022; Appendix 4 in ESM); As, Pb, Cu, Ni and Cr were significantly higher than the average limits detected in the same species of South Australia (Kuppusamy et al. 2017; Appendix 4 in ESM). The mean concentrations in *A. cepa* (onion) produced in the SB region (data of this work; Appendix 4 in ESM) were lower for: Cd and Pb than those from the Kosovo thermoindustrial zone (Zeneli et al. 2013); Cd than those from the Liaoning Province (China; Li et al. 2016) and South Australia (Kuppusamy et al. 2017); As than those found in Vale do Siran (Pakistan; Tahir et al. 2022).

The maximum concentrations of Cu, Zn, Ni, and Cr detected in cheese (produced with *B. taurus* milk) from the SB region were significantly higher than in the other areas of Rio de Janeiro (Azcue et al. 1988; Appendix 4 in ESM). In the SB region’s *B. taurus* cheese, the maximum Hg levels found were higher than those investigated in Algeria (Chebli et al. 2024; Appendix 4 in ESM), but for Cd and Pb they were lower than those from other places in the Rio de Janeiro State (Azcue et al. 1988) and Algeria (Chebli et al. 2024, Appendix 4 in ESM).

In *C. annum* (pepper) from the SB region (Appendix 4 in ESM), the average As contents were similar to those produced in industrial areas of China (Li et al. 2016; however, the maximum As values were lower than those from Turkey (Tokalıoğlu et al. 2019; Appendix 4 in ESM). Meanwhile, in *C. annum* (pepper) of the SB region (Appendix 4 in ESM) the average and/or maximum concentrations of: Cu, Cd, Cr, and Hg were lower than those produced in China (Zheng et al. 2007; Li et al. 2016), Turkey (Tokalıoğlu et al. 2019) and Bangladesh (Mizan et al. 2023); Zn were higher than those produced in Liaoning Province (China; Li et al. 2016) and Turkey (Tokalıoğlu et al. 2019) but were lower than in this species in Bangladesh (Mizan et al. 2023; Appendix 4 in ESM); Ni were higher than those of Bangladesh (Mizan et al. 2023) and Turkey (Tokalıoğlu et al. 2019).

The maximum concentrations of Cr in *C. frutescens* (chili) of the SB region were higher than those surrounding the roads of Nigeria (Olayinka and Ipaiyeda 2011; Appendix 4 in ESM). However, the maximum Pb values in this species in Nigeria were less significant than those in the SB region (Olayinka and Ipaiyeda 2011; Appendix 4 in ESM).

*Manihot esculenta* (yam) from the SB region had higher PTE concentrations (Cd, Pb, Cu, Zn, Ni, and Cr) than those cultivated in volcanic soils from the Canary Islands (Spain; Luis-González et al. 2015; Appendix 4 in ESM), despite these regions being directly influenced by the emission of toxic gases and metals from geogenic sources (Nriagu and Becker 2003; Doelsch et al. 2006).

The maximum levels in *Cucurbita moschata* (pumpkin) of the SB region of Cu, Zn, Cr, and Ni were higher than those detected in *Cucurbita sp.* collected in the other areas of Rio de Janeiro (Azcue et al. 1988) and Bangladesh (Mizan et al. 2023; Appendix 4 in ESM), and lower than those produced in Bangladesh (Dhaka; Mizan et al. 2023; Appendix 4 in ESM). The mean Hg values in this species of the SB region were similar to those in Liaoning Province, China (Zheng et al. 2007; Appendix 4 in ESM).

The maximum As concentrations detected in *G. gallus domesticus* (chicken) eggs from the SB region are higher than those of Algeria (Zergui et al. 2023), China (Zhao et al. 2021) and Italy (Esposito et al. 2016; Appendix 4 in ESM). On the other hand, Cd reached higher maximum concentrations in eggs of *G. gallus domesticus* in the regions mentioned above than in the SB region (Esposito et al. 2016; Zhao et al. 2021; Zergui et al. 2023). Copper, Zn, Ni, Co, Cr, and Hg in chicken eggs from SB also showed higher concentrations than in other studies (Esposito et al. 2016; Zhao et al. 2021; Zergui et al. 2023).

The maximum concentration contents in *L. sativa* (lettuce) from SB region of: Cu, Zn, Ni and Cr exceeded those from the other areas of Rio de Janeiro State (Azcue et al. 1988) and in Aseer, SW of Saudi Arabia (Oteef et al. 2015; Appendix 4 in ESM); Pb were also higher than those produced in Aseer (Oteef et al. 2015; Appendix 4 in ESM) but were lower than those found in other regions of Rio de Janeiro (Azcue et al. 1988; Appendix 4 in ESM).

*Musa acuminata* Colla (banana) revealed higher mean values of all analyzed PTEs (As, Cd, Pb, Cu, Zn, Ni, Co, Cr, and Hg) than those found in other parts of the world, such as South Korea and South Australia (Habte et al. 2017; Kuppusamy et al. 2017; Appendix 4 in ESM).

Similarly, the *P. vulgaris* (bean) produced in the SB region showed higher mean concentrations of Cd, Pb, Cu, Zn, Cr and Hg than those found in other arable areas from the Rio de Janeiro State (Azcue et al. 1988), China (Zheng et al. 2007; Li et al. 2016) and Pakistan (Siran Valley; Tahir et al. 2022) but for As they were lower than those found in the mentioned regions (Appendix 4 in ESM).

The maximum concentrations of Cd, Cu, Zn, Ni and Cr in *S. lycopersicum* (tomato) of the SB region exceeded those from the other areas of Rio de Janeiro (Azcue et al. 1988) and Saudi Arabia (Oteef et al. 2015; Appendix 4 in ESM), but were lower for Pb in other regions of Rio de Janeiro (Azcue et al. 1988) and Saudi Arabia (Aseer; Oteef et al. 2015; Appendix 4 in ESM).

In *S. melongena* (eggplant) of SB region, the maximum concentrations of: Cu were higher than those of Mamfe region (Cameroon; Agbor et al. 2024) and Dhaka (Bangladesh; Mizan et al. 2023; Appendix 4 in ESM); Zn were higher than those of in Mamfe (Cameroon; Agbor et al. 2024) but lower than those found in Dhaka (Bangladesh; Mizan et al. 2023; Appendix 4 in ESM); Cd, Pb, Ni and Cr were lower than in Cameroon (Mamfe; Agbor et al. 2024) and Bangladesh (Dhaka; Mizan et al. 2023; Appendix 4 in ESM).

*Solanum tuberosum* (potato) from the SB region (Appendix 4 in ESM) showed higher maximum concentrations of Cu, Zn, Cr and Pb than those in Peru (Andes; Orellana-Mendoza et al. 2024) and Kosovo (Zeneli et al. 2013), but displayed lower Cd concentrations than those in Kosovo (Zeneli et al. 2013).

### Enrichment and dispersion of PTEs in agricultural products

Elements As and Co from Subgroup 1.1 of the CA of Fig. 5. Arsenic appears in high concentrations in some of the analyzed species (Fig. 4; Appendix 1 in ESM). Very high levels of arsenic (As) were detected in the ore, sediments, and soils of the SB region (Magalhães and Pfeiffer 1995; Magalhães et al. 2001; Damasceno et al. 2024a). In the study area, As is typically associated with metallurgical processes and the use of As_2_O_3_ (Magalhães and Pfeiffer 1995; Magalhães et al. 2001). Cobalt is frequently found as a byproduct of metal alloy processing (Mudd et al. 2013). The region surrounding Sepetiba Bay has housed an industrial hub since the 1970s (Morales et al. 2019; Ribeiro et al. 2015). Thus, the enrichment of As and Co may be induced by anthropogenic activities in the SB region.

Elements Hg, Ni and Cr from Subgroup 1.2 of the CA of Fig. 5 exhibit distinct chemical characteristics compared to the previously mentioned group. In aqueous environments, particularly acidic ones, they can become more mobile (Kicińska et al. 2022). Mercury and Cr may be related to natural sources. Mercury is present in the Itatiaia and Tanguá Alkaline Complexes (Pires et al. 2014). Furthermore, relatively high concentrations of Hg were found in SB sediments dated to between 9000–8000 calibrated BP, which were exposed to subaerial processes and later submerged following the inundation of the external sector of the SB during the early to mid-Holocene (Castelo et al. 2021a). This suggests the presence of relatively high Hg contents thousands of years before the region’s industrialization, indicating the presence of natural sources of Hg in the area associated with the history of hydrothermalism that has occurred there and the interaction of the water table with the overlying sedimentary environment (Castelo et al. 2021a).

Chromium may also be related to local geology. The metasedimentary rocks in the region, including the calcium-alkaline orthogneisses of the Quirino Unit from the Paraíba do Sul Complex, were reported to have high Cr contents (Valladares 1996; Valladares et al. 1997). Niquel may occur alongside iron and manganese mineral phases and ferrous sulfides in the study area (Muniz et al. 2004). Thus, the enrichment of these elements can be considered to be influenced by geogenic processes in the study area.

Group 2 of CA includes metals, such as Zn, Cu, Pb, and Cd (Fig. 5), that are most commonly found in relatively high concentrations in the sediments of SB (Barcellos et al. 1991; Ribeiro et al. 2013; Silva et al. 2022a; Saibro et al. 2023; Damasceno et al. 2024a). In addition, the significant positive correlations between Zn, Cd, Pb, and Cu (Appendix 4 in ESM) suggest a strong relationship with mineral processing activities (Sun and Chen 2018), as these elements naturally co-occur with sulfides in mineral phases (Smuda et al. 2007; Zhou et al. 2020). These metals are widely used in the industry, one of Brazil’s leading export economies, particularly in the Sepetiba Bay area (Machado and Figueirôa 2022). Araújo et al. (2017) associate these metals with the activity of Ingá Mercantil mining company waste that labored in Madeira Island (Sepetiba Bay) until 1985. The Ingá Mercantil operated for 27 years and carried out metal alloy processing activities in the SB region (Ribeiro et al. 2015). Despite its inactivity, the metallurgy company was responsible for successive tailings dam failures, contaminating Sepetiba Bay and the surrounding areas with PTEs (Lacerda et al. 1987; Gomes et al. 2009; Paraquetti et al. 2004; Rodrigues et al. 2020).

Although there are no studies with soil samples specifically from the cultivation sites of the food products analyzed in this study, recent sediment data from Sepetiba Bay show high levels of metals such as Cd, Zn, Cu, and Pb in the Coroa Grande mangrove, where the mouth of the Mazomba River is located; this river crosses the area where the agricultural products are produced (Rodrigues et al. 2025; Fig. 1).

Stable isotope analyses of Zn and Pb confirm the anthropogenic origin of these PTEs in Sepetiba Bay sediments (Cunha et al. 2009; Cunha et al. 2011). In this regard, Pinto et al. (2019) evaluated the degree of accumulation of these PTEs in sediments dated between approximately 1,700 and 2,300 years before present, collected on the northern shore of the bay, and found relatively low concentrations. Thus, this suggests that the high levels currently found (Souza et al. 2021; Damasceno et al. 2024a; Rodrigues et al. 2025) are a direct consequence of anthropogenic activity, particularly industrial, that has occurred since the second half of the twentieth century (Pinto et al. 2019).

In addition to sedimentary geochemical evidence, studies on sediments and native vegetation from the Sepetiba Bay mangroves also indicate the influence of PTE enrichment, particularly Zn and Cu, as evidenced by leaves collected at the mouth of the Mazomba River (Fonseca et al. 2013; Victório et al. 2020). An environmental monitoring study in the Guandu River basin also identified significant concentrations of PTEs (Cu, Mn, Pb, Zn, Ni, and Cr) in water, sediment, and soil samples, reinforcing the scenario of PTE enrichment in the environment (Lopes et al. 2016).

Meanwhile, even though the producers have organic food certification and use only natural soil correction and defense techniques, industrial activities in the region, a railroad located close to the family farm (≈5 km) transports iron ore and fertilizers to the port of Itaguaí (Figs. 1, 2), which contains significant levels of these elements (Damasceno et al. 2024a). Studies conducted in the mangrove of Ilha da Madeira (Itaguaí Port) indicate that the tailings deposit is the primary source contributing to contamination by Cd, Pb, Cu, Cr, Hg, Ni, and Zn in Sepetiba Bay (Fonseca et al. 2013).

Significant concentrations of Zn and Cd in the surface sediments, suspended material, and the water column of SB were studied by Barcellos (1995). The dispersion of Hg in sediments associated with organic complexes was evaluated by Lacerda et al. (2001) in the same region. Lead isotopes confirmed contamination, especially in SB's eastern and northeastern sectors (Morales et al. 2019). Over the years, a degree of attenuation in PTE presence has been identified in previously marked contamination hotspots, suggesting the dispersion of PTEs to other parts of the SB (Lacerda et al. 2004; Rodrigues et al. 2020). The increased industrialization in the region promoted the accumulation of PTEs, with a significant upward trend in the concentrations of these elements being detected (Souza et al. 2021; Damasceno et al. 2024a). According to Castelo et al. (2021a), Silva et al. (2022b), and Damasceno et al. (2024b), the sediments of the inner area of Sepetiba Bay have high ecological risk due to high concentrations of PTEs (Castelo et al. 2021a; Silva et al. 2022b; Damasceno et al. 2024b). Considerable PTE contents, such as Cd, Pb, Cu, Cr, Hg, Ni, and Zn, were detected in sediments and mangrove plants (Fonseca et al. 2013; Victório et al. 2020). PTE contamination loads in SB directly impact benthic species (Castelo et al. 2021b). Several animals, such as oysters and fish from the SB region, have also evidenced the negative impacts of PTE accumulation (Lima Junior et al. 2002; Lacerda et al. 2006).

Given the mobility of PTEs, it is plausible that the metal content found in the food items may also be associated with interactions with aqueous environments, likely from the Piranema aquifer. This aquifer belongs to the interconnected aquifer system of Sepetiba Bay. The food cultivation zone is naturally irrigated by the Mazomba River (Fig. 1), which originates from the Mazomba Mountain in the Serra do Mar. The Neosols (Fig. 2) are directly associated with the surrounding rocks. Despite the essential mineralogy of the SB region rocks being characterized by considerable silica (SiO_2_) content, André et al. (2021) also found concentrations of Fe, Zn, Cr, Co, Ni, Cu, and Pb, for example, in the Orthogranulites of Ribeirão das Lajes. Neosols have specific properties, such as sandy texture and porosity, which facilitate metal mobility in the soil and contact with the water table (Santos et al. 2018).

The transfer of PTEs present in soils to plants depends on a combination of factors, including soil characteristics, composition, pH, redox conditions, organic matter content, competitive interactions between cations, natural irrigation, and more (Adamo et al. 2014; Palansooriya et al. 2020). In this sense, the permeability of soils such as Neosols enhances the transport and dispersion of metals through fluids (Hellweg et al. 2005). Certain plant species can absorb metals more (Adamo et al. 2014).

The calculated Pollution Risk Potential Indices revealed that foods such as eggs (white > shell > yolk), *Capsicum chinense* (pout pepper), *Arachis hypogaea* (peanut), *Lactuca sativa* (lettuce), *Phaseolus vulgaris* (red and white bean), cheese (produced with milk from *Bos taurus*), *Vigna unguiculata* (cowpea) made in the Sepetiba Bay region are significantly polluted (HI > 1). The elements that contributed the most to the hazard level were As and Cr, as indicated by the HQ values (Appendix 3 in ESM; Table 4).

The HQ values (Adhikari et al. 2022; Sikakwe et al. 2024) in this study reveal that the analyzed elements exhibit varying degrees of toxicity. Arsenic and Cr may present a greater potential risk, as even at low concentrations, they can cause health problems (Jaishankar et al. 2014). Thus, it is essential to note that the relatively high HI values (> 1) indicate a long-term carcinogenic risk in adults (Ogunkunle et al. 2022; Table 4).

In this study, the highest levels of PTEs were found, in general, in root-based foods (Figs. 4, 7; Fig. S1). Metals are mainly absorbed through plant roots (Cobb et al. 2000) and can be distributed via the apoplast (the spaces between cells; Gupta et al. 2019). Transport to other parts of the plant is facilitated by the vascular tissues (xylem and phloem), which move raw sap and processed sap, and enable the mobility of metals in solution (Page and Feller 2015; Cao et al. 2020). In this context, it is expected that greater enrichment of PTEs will be found in the roots of the analyzed foods from the SB region.

The toxic effects of PTEs, resulting from their transfer from the environment to the roots, were studied by Altunkaynak et al. (2023). Through laboratory tests involving the growth of *A. cepa* (Onion) in a medium contaminated with metallic substances, numerous anomalies in plant cells were confirmed (Sabeen et al. 2020; Altunkaynak et al. 2023). As a result, the levels of PTEs detected in fruits and seeds are lower than those found in roots, stems, and leaves (Cobb et al. 2000). This is because plants have defense mechanisms in reproductive organs for the biological protection of the species (Xun et al. 2017). Meindl and Ashman (2015) revealed that Ni levels in the reproductive organs of plants deter pollinating insects, such as bees, thereby directly affecting the perpetuation of the species.

However, analyses carried out on fruits by Yumuşakbaş et al. (2025) revealed high levels of PTEs (Cd and Cu), corroborating the localized accumulation of these elements in the parts of the plant surrounding the SB region. The fruits and seeds analyzed in the SB region revealed considerably high concentrations of PTEs (Fig. 8). In this sense, metals that have high mobility, such as Zn, Ni, Cd, and As can overcome the plant's biological protection barriers more easily (Page and Feller 2015). On the other hand, elements with low to moderate mobility, such as Pb, Cu, Co, Cr, and Hg, in acidic environments facilitate their translocation from soils to plants. It should be noted that Cr and Hg, in the more mobile stages, reach more toxic phases (Richard and Bourg 1991; Reis et al. 2010; Table 4).

Chicken eggs are considered one of the most complete foods in human nutrition (Surai and Sparks 2001). Proteins are distributed throughout all parts of chicken eggs. However, the egg white makes up 60% of the total and has a greater capacity to retain chemical compounds and proteins (Mine et al. 2002; Kovacs-Nolan et al. 2005). The various parts of the analyzed eggs (eggshells, whites and yolks) showed dissimilar metal concentrations (Fig. 6). In this work, the egg whites revealed significantly higher average levels of As, Pb, and Hg than the other parts of the same product. Egg white (albumen*)* is considered a protective barrier, the site of the most significant nutrient absorption (Obianwuna et al. 2022).

However, egg yolks showed comparatively higher mean values of Cu, Zn, and Cr than the other parts of the eggs in this study. Studies conducted in Lahore, Pakistan, have revealed a more significant accumulation of PTEs in egg yolks compared to egg whites (Kabeer et al. 2020). The eggshells of the SB region, when compared to the other analyzed parts, revealed relatively high concentrations of Ni and Co compared to whites and yolks. In the case of animal products, other factors, such as the chemical components of the food formula, can increase the accumulation of PTEs in this type of food (Zhang et al. 2012).

## Conclusion

The present study examined the contamination by PTEs in agricultural products grown in the surroundings of Sepetiba Bay (SE), Rio de Janeiro State (SE Brazil). Copper and Zn reached the highest significant concentrations in the studied foods (> 100 mg kg^−1^). Copper, Zn, Ni, Co, and Cr were detected in all analyzed foods, especially in roots > fruits > seeds. The concentrations of metals exceeded the limits defined by the regulatory agencies (ANVISA and FAO/China) in all the analyzed foods. However, not all the samples of each type of food showed the same pattern of metal enrichment. Spatial differences in the degree of metal contamination of soils and irrigation water, as well as the high porosity, permeability, and chemical properties of soils, and their relationship with groundwater tables in the region surrounding Sepetiba Bay, can contribute to changes in the geochemical behavior of metals and consequent transfer to food cultivated in this area. The direct connection between the Mazomba River and the investigated agricultural area, as well as the typical flooding characteristics of the region surrounding the study site, raise the hypothesis that potential contaminants transported along the basin may influence the presence of potentially toxic trace elements in agricultural products, which, although organic, may be contaminated by Zn > Cu > Ni > Cr > As > Pb > Co > Cd > Hg.

The statistical analyses enabled the grouping of elements based on both quantity and geochemical behavior and/or source. All the PTEs analyzed were widely detected in the sediments of Sepetiba Bay. The Cluster analysis (CA) grouped the metals according to their primary sources of enrichment, which were either geogenic or anthropogenic.

Thus, it can be concluded that food cultivated in the surrounding region of Sepetiba Bay may pose a potential risk of contamination by PTEs, especially by As and Cr (due to their toxicity and high concentration levels), for the local population. Among the food items analyzed, those with the most significant risk of generating health problems for the population due to the degree of Cr and/or As contamination were for instance *Allium cepa* (onion), *Ananas comosus* (pineapple), *Bos taurus* cheese, *Capsicum annum* (green, red, yellow pepper), *Capsicum chinense* (pout and yellow pout pepper), *Cucurbita maxima* (pumpkin sergipana), *Cucurbita moschata* (mini pumpkin and pumpkin barbara), *Daucus carota* (Carrot), *Gallus gallus domesticus* (chicken egg yolk and white), *Lactuca sativa* (lettuce), *Manihot esculenta* (cassava), *Musa acuminata* (banana), *Phaseolus lunatus* (fava beans), *Phaseolus vulgaris* (red, green and white beans), *Solanum lycopersicum* (tomato), *Solanum melongena* (eggplant), and *Vigna unguiculata* (String beans and cowpeas).

Frequent consumption of food contaminated by PTEs may lead to metal accumulation in human organs. Heavy metal contamination in humans is considered a primary cause of various health issues, damaging vital cells and tissues.

There is a lack of information on PTEs contamination in food in Rio de Janeiro State and other areas in Brazil. Considering the importance of food safety and the significant impact of bioaccumulation of potentially toxic elements in food on public health, detailed studies on this topic and the development of strategies to mitigate existing pollution problems are necessary.

## Conflicts of interest

The authors declare that there are no conflicts of interest.

## Data availability:

The data supporting this work are available in tables and as supplementary materials.

- Funding—Financing was received from:

Virginia Martins: Fundação Carlos Chagas Filho de Amparo à Pesquisa do Estado do Rio de Janeiro—FAPERJ (Edital Projetos Temáticos no Estado do Rio de Janeiro- 2021, process # E-26/211.278/2021). FAPERJ (process # E-26/200.333/2023). Conselho Nacional de Desenvolvimento Científico e Tecnológico of Brazil, CnPQ (process # 305,719/2023–8). Graziele Reis: Coordenação de Aperfeiçoamento de Pessoal de Nível Superior (CAPES; process # E- 88887.718324/2022-00).

-Ethical Approval—The authors followed the ethical standards.

-Consent to Participate—The authors have consent to participate.

-Consent to Publish—The authors have consent to publish.

## Supplementary Information

Below is the link to the electronic supplementary material.

Supplementary file 1 (XLSX 9 KB)

Supplementary file 2 (DOCX 127 KB)

Supplementary file 3 (DOCX 32 KB)

Supplementary file 4 (XLSX 39 KB)

Supplementary file 5 (XLSX 23 KB)

Supplementary file 6 (XLSX 31 KB)

Supplementary file 7 (XLSX 32 KB)
