# Supplementary material for: Contamination by Potentially Toxic Elements (PTEs) in Agricultural Products Grown Around Sepetiba Bay, Rio de Janeiro State (SE Brazil)
Source: Arch Environ Contam Toxicol. 2025 Aug 6;89(2):195–220. doi: 10.1007/s00244-025-01143-8 (PMC12414040; doi:10.1007/s00244-025-01143-8)
Supplement: Supplementary file 2 — (DOCX 127 KB) [file 244_2025_1143_MOESM2_ESM.docx]

|  |
| --- |

Supplementary Figure 1. Concentration of metals in the analyzed food items. The acronyms used are identified in Appendix 1 and in the following table:

| **Species** | **Food Item** | **Acronym** | **Species** | **Food Item** | **Acronym** |
| --- | --- | --- | --- | --- | --- |
| *Allium cepa* | Onion | Onion1 | *Gallus gallus domesticus* (chicken) | Egg yolk2 | Y.Egg.2 |
| *Allium cepa* | Onion | Onion2 | *Gallus gallus domesticus* (chicken) | Egg white1 | W.Egg,1 |
| *Allium cepa* | Onion | Onion3 | *Gallus gallus domesticus* (chicken) | Egg white2 | W.Egg.2 |
| *Ananas comosus* | Pineapple | Pineapple | *Gallus gallus domesticus* (chicken) | Egg Shell1 | S.Egg.1 |
| *Arachis hypogaea* | Peanut | Peanut | *Gallus gallus domesticus* (chicken) | Egg Shell2 | S.Egg.2 |
| *Bos taurus* | Cheese | Cheese | *Ipomoea batatas* | Sweet potato | S.Potato |
| *Capsicum annum* | Green pepper | G.Pepper | *Lactuca sativa* | Lettuce | Lettuce |
| *Capsicum annum* | Red pepper | R.Pepper | *Manihot esculenta Crantz* | Cassava | Cassava1 |
| *Capsicum annum* | Yellow pepper | Y.Pepper | *Manihot esculenta Crantz* | Cassava | Cassava2 |
| *Capsicum chinense* | Pout pepper | P.Pepper | *Musa acuminata Colla* | Banana | Banana1 |
| *Capsicum chinense* | Yellow Pout pepper | Y.P.Pepper | *Musa acuminata Colla* | Banana | Banana2 |
| *Capsicum frutescens* | Green Chili pepper | G.Chili | *Persea americana* | Avocado | Avocado |
| *Capsicum frutescens* | Red Chili pepper1 | R.Chili1 | *Phaseolus lunatus* | Fava Bean | F.Bean |
| *Capsicum frutescens* | Red Chili pepper2 | R.Chili2 | *Phaseolus vulgaris* | Red Bean | R.Bean |
| *Colocasia esculenta* | Yam | Yam | *Phaseolus vulgaris* | Green Bean | G.Bean |
| *Cucurbita maxima* | Pumpkin Sergipana | Pumpkin.S | *Phaseolus vulgaris* | White Bean | W.Bean |
| *Cucurbita moschata* | Mini pumpkin | M.Pumikin1 | *Solanum lycopersicum* | Tomato | Tomato |
| *Cucurbita moschata* | Mini pumpkin | M.Pumpkin2 | *Solanum melongena* | Eggplant | Eggplant |
| *Cucurbita moschata* | Pumpkin Barbara | Pumpkin.B | *Solanum tuberosum* | Potato | Potato |
| *Daucus carota* | Carrot | Carrot | *Vigna unguiculata* | String Bean | S.Bean |
| *Gallus gallus domesticus* (chicken) | Egg yolk1 | Y.Egg.1 | *Vigna unguiculata* | Fradinho Bean or cowpea | Fr.Bean |
