# Supplementary material for: Contamination by Potentially Toxic Elements (PTEs) in Agricultural Products Grown Around Sepetiba Bay, Rio de Janeiro State (SE Brazil)
Source: Arch Environ Contam Toxicol. 2025 Aug 6;89(2):195–220. doi: 10.1007/s00244-025-01143-8 (PMC12414040; doi:10.1007/s00244-025-01143-8)
Supplement: Supplementary file 3 — (DOCX 32 KB) [file 244_2025_1143_MOESM3_ESM.docx]

| **Vegetal** | **As** | **Cd** | **Pb** | **Cu** | **Zn** | **Ni** | **Cr** | **Hg** |
| --- | --- | --- | --- | --- | --- | --- | --- | --- |
| Onion1 | 🔴 | 🟡 | 🟢 | 🟢 | 🔴 | 🟡 | 🔴 | 🔴 |
| Onion2 | 🟢 | 🟢 | 🔴 | 🟢 | 🟢 | 🟢 | 🔴 | 🟢 |
| Onion3 | 🔴 | 🟢 | 🟡 | 🟢 | 🔴 | 🟢 | 🔴 | 🔴 |
| Pineaple | 🟢 | 🔴 | 🟢 | 🟡 | 🔴 | 🔴 | 🔴 | 🔴 |
| Peanut | 🟡 | 🟡 | 🟢 | 🔴 | 🔴 | 🟡 | 🟢 | 🟡 |
| Cheese | 🟡 | 🟢 | 🔴 | 🔴 | 🟡 | 🟡 | 🟡 | 🟢 |
| Green pepper | 🟢 | 🟡 | 🔴 | 🔴 | 🟢 | 🟢 | 🔴 | 🟢 |
| Red pepper | 🔴 | 🟡 | 🟢 | 🟡 | 🟢 | 🟢 | 🟡 | 🟡 |
| Yellow pepper | 🟢 | 🟡 | 🔴 | 🔴 | 🟡 | 🔴 | 🔴 | 🔴 |
| Pout pepper | 🟢 | 🔴 | 🔴 | 🟡 | 🔴 | 🟢 | 🟢 | 🟡 |
| Yellow Pout pepper | 🟢 | 🟢 | 🔴 | 🔴 | 🟢 | 🟡 | 🟡 | 🔴 |
| Green Chilli pepper | 🟢 | 🔴 | 🟡 | 🟢 | 🔴 | 🔴 | 🟡 | 🔴 |
| Red Chilli pepper1 | 🟡 | 🟡 | 🟢 | 🟢 | 🔴 | 🟡 | 🟡 | 🟢 |
| Red Chilli pepper2 | 🟢 | 🟡 | 🟢 | 🔴 | 🟡 | 🟢 | 🔴 | 🟡 |
| Yam | 🔴 | 🔴 | 🟡 | 🔴 | 🟢 | 🟢 | 🟢 | 🔴 |
| Pumpikin Sergipana | 🟢 | 🔴 | 🟢 | 🟡 | 🔴 | 🔴 | 🟢 | 🟡 |
| Mini pumpkin | 🟢 | 🟢 | 🟡 | 🟡 | 🟢 | 🟡 | 🟢 | 🔴 |
| Mini pumpkin | 🟢 | 🔴 | 🟢 | 🟡 | 🔴 | 🟡 | 🔴 | 🔴 |
| Pumpukin Barbara | 🟢 | 🟢 | 🟢 | 🟢 | 🟡 | 🟢 | 🟢 | 🔴 |
| Carrot | 🟡 | 🟡 | 🔴 | 🔴 | 🟢 | 🟡 | 🟢 | 🔴 |
| Egg yolk | 🟢 | 🟡 | 🟢 | 🟡 | 🟢 | 🟢 | 🟡 | 🟡 |
| Egg yolk | 🟡 | 🟢 | 🟢 | 🟢 | 🔴 | 🟢 | 🟡 | 🟡 |
| Egg white | 🟡 | 🟢 | 🟡 | 🟡 | 🔴 | 🔴 | 🟡 | 🔴 |
| Egg white | 🔴 | 🟡 | 🟢 | 🟢 | 🟡 | 🟢 | 🔴 | 🟡 |
| Egg Shell | 🟢 | 🟢 | 🔴 | 🟡 | 🟡 | 🔴 | 🟡 | 🟡 |
| Egg Shell | 🔴 | 🔴 | 🟢 | 🟡 | 🔴 | 🟢 | 🟡 | 🟢 |
| Sweet potato | 🔴 | 🟡 | 🟢 | 🔴 | 🔴 | 🔴 | 🔴 | 🔴 |
| Lettuce | 🔴 | 🔴 | 🟢 | 🟡 | 🟡 | 🟢 | 🔴 | 🟡 |
| Cassava1 | 🔴 | 🟡 | 🔴 | 🟢 | 🔴 | 🟡 | 🔴 | 🟡 |
| Cassava2 | 🔴 | 🟡 | 🟡 | 🟡 | 🔴 | 🟡 | 🔴 | 🟢 |
| Banana1 | 🟢 | 🟡 | 🔴 | 🟡 | 🔴 | 🔴 | 🟢 | 🔴 |
| Banana2 | 🔴 | 🟡 | 🟡 | 🔴 | 🟢 | 🟡 | 🟡 | 🟢 |
| Avocado | 🟡 | 🔴 | 🔴 | 🔴 | 🟡 | 🟡 | 🟡 | 🟡 |
| Fava Bean | 🔴 | 🟢 | 🔴 | 🟡 | 🔴 | 🟢 | 🔴 | 🟢 |
| Red Bean | 🟡 | 🔴 | 🔴 | 🔴 | 🔴 | 🟡 | 🟢 | 🟢 |
| Green Bean | 🔴 | 🟡 | 🟢 | 🟢 | 🟢 | 🟢 | 🟢 | 🟢 |
| White Bean | 🟡 | 🔴 | 🟢 | 🟡 | 🟡 | 🔴 | 🔴 | 🟢 |
| Tomato | 🔴 | 🔴 | 🟡 | 🟡 | 🟡 | 🟢 | 🟢 | 🔴 |
| Eggplant | 🔴 | 🟢 | 🟢 | 🟡 | 🟢 | 🟢 | 🔴 | 🔴 |
| Potato | 🟡 | 🟢 | 🔴 | 🟢 | 🟢 | 🟡 | 🟢 | 🟡 |
| String Bean | 🔴 | 🔴 | 🔴 | 🔴 | 🟡 | 🟡 | 🔴 | 🔴 |
| Fradinho Bean | 🔴 | 🟡 | 🔴 | 🟡 | 🟢 | 🔴 | 🟡 | 🟡 |

Supplementary Figure 2: Heatmap highlighting food items that exceeded regulatory limits (red), those requiring attention (yellow), and those within acceptable levels (green).
